# Poultry Response to Heat Stress: Its Physiological, Metabolic, and Genetic Implications on Meat Production and Quality Including Strategies to Improve Broiler Production in a Warming World

**DOI:** 10.3389/fvets.2021.699081

**Published:** 2021-07-23

**Authors:** Ali H. Nawaz, Kwaku Amoah, Qi Y. Leng, Jia H. Zheng, Wei L. Zhang, Li Zhang

**Affiliations:** ^1^College of Coastal Agricultural Sciences, Guangdong Ocean University, Zhanjiang, China; ^2^Aquatic Animals Precision Nutrition and High-Efficiency Feed Engineering Research Center of Guangdong Province, Zhanjiang, China

**Keywords:** heat stress, poultry, meat production, meat quality, muscle development

## Abstract

The continuous increase in poultry production over the last decades to meet the high growing demand and provide food security has attracted much concern due to the recent negative impacts of the most challenging environmental stressor, heat stress (HS), on birds. The poultry industry has responded by adopting different environmental strategies such as the use of environmentally controlled sheds and modern ventilation systems. However, such strategies are not long-term solutions and it cost so much for farmers to practice. The detrimental effects of HS include the reduction in growth, deterioration of meat quality as it reduces water-holding capacity, pH and increases drip loss in meat consequently changing the normal color, taste and texture of chicken meat. HS causes poor meat quality by impairing protein synthesis and augmenting undesirable fat in meat. Studies previously conducted show that HS negatively affects the skeletal muscle growth and development by changing its effects on myogenic regulatory factors, insulin growth factor-1, and heat-shock proteins. The focus of this article is in 3-fold: (1) to identify the mechanism of heat stress that causes meat production and quality loss in chicken; (2) to discuss the physiological, metabolic and genetic changes triggered by HS causing setback to the world poultry industry; (3) to identify the research gaps to be addressed in future studies.

## Introduction

The increasing world population demands a more efficient food production system since the global food shortage issue keeps on rising. The poultry sector is noted to make a considerable contribution to global nutrition and food security, which helps in the provision of cheap protein, essential micronutrients, and energy to humans (1). Poultry, owing to their short production cycles and having the potential of converting wide ranges of agricultural food waste and by-products into eggs and meat edible for humans. Poultry meat production have been reported to increase from 120.5 MMT (million metric tons) in 2017 to 122.5 MMT in 2018 (2). FAO (3) also estimated its production to reach 137 MMT in 2020, with growth being anticipated in China, Britain, the EU, Mexico, and Brazil, suggesting the poultry industry's hidden potentials.

**Figure 1:**
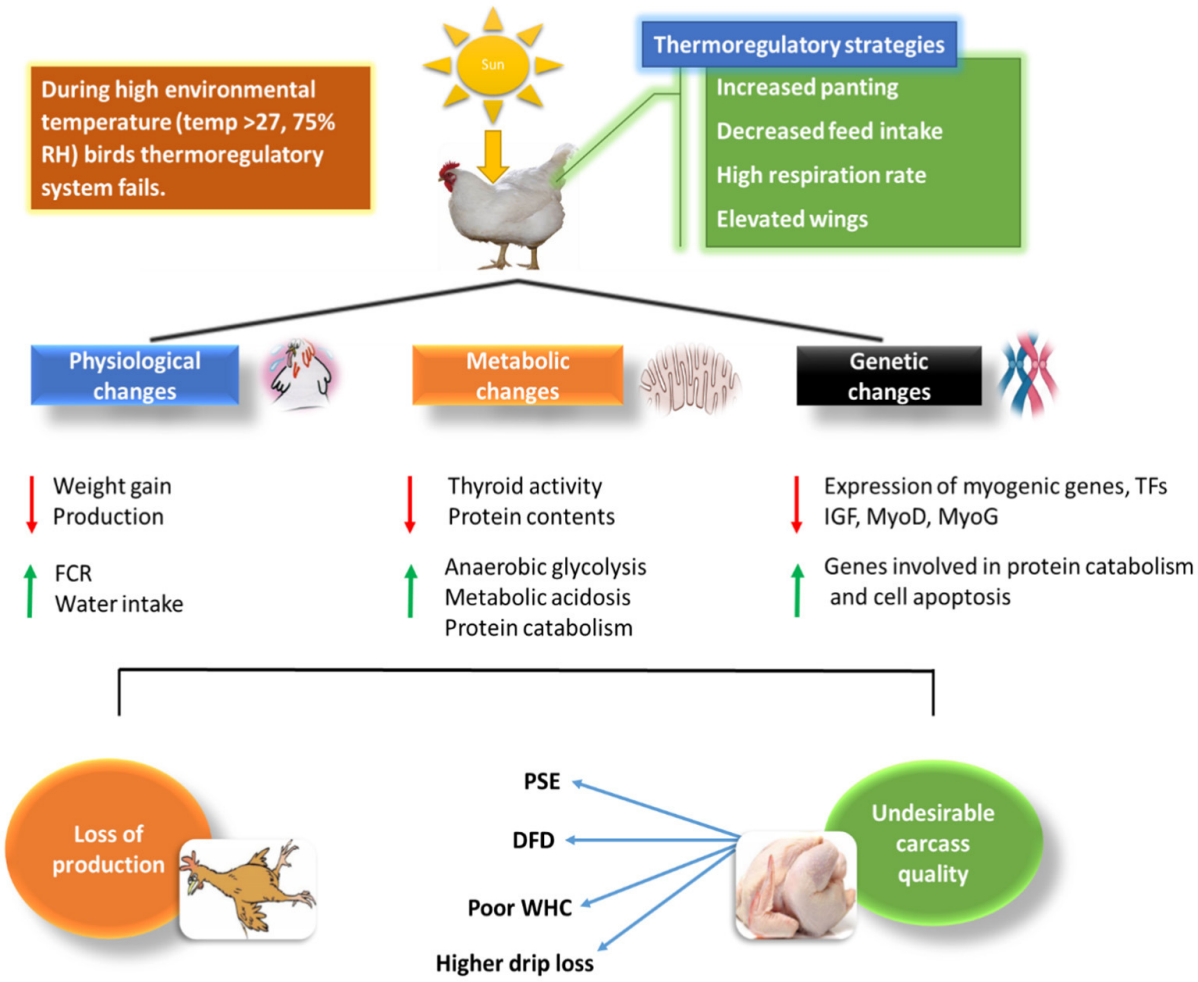
Relationship of HS with physiological and biochemical changes in chicken and how it affects broiler chickens' meat quality.

Recently, there has been a remarkable escalation in global environmental temperature, which poses serious implications to the farming sector in both tropical and subtropical regions of the world. A gradual increase in ambient temperature affects all living organisms (4, 5). In living organisms, if the temperature exceeds the normal range (thermo-neutral zone), it disturbs the normal physiological functioning and induces cell injury. Usually, high ambient temperature leads to stress associated problems such as production losses, metabolic changes, growth depression, and poor efficiency (6, 7). In temperate regions of world the high ambient temperature during the summer season often proves disastrous for poultry farming as thermal stress induced by extremely high temperatures is responsible for massive economic losses to poultry industry. According to a report, the U.S. livestock production industry suffers a severe loss of $1.69 to $2.36 billion because of high environmental temperature; out of which poultry industry accounts the loss of $128 to $165 million (8). Heat stress (HS) is widely classified into acute heat stress (AHS), which is the intense environmental temperature for a brief period and chronic heat stress (CHS) characterized by high temperature for a longer duration. Unluckily, both AHS and CHS challenge the genetic, nutritional, pharmaceutical, and management developments made by the animal farming industries that cause a considerable drop in production, proving to be one of the major hurdles to achieve efficient livestock farming in many regions of the world (9, 10). Chronic heat stress has permanent damaging effect on the broiler chicken, if heat stress persists for longer period of time it increases fat content and damages the muscle portion of chicken unlike acute heat stress. Apart from duration of heat stress, the extent of production damage is also dependent on the intensity of heat stress (11). Harmful consequences of heat exhaustion (temperature exceeds beyond thermo-neutral zone and animal no more able to regulate body temperature) in animal farming would become more challenging as temperature keeps rising due to global warming. Climate change due to global warming is becoming more relevant these days, especially for the chicken meat industry (12, 13). The broiler industry faces the challenge of HS, which increases production cost and severely damages the meat quality due to poultry's susceptibility to heat because of their rapid metabolic rate and high growth. Metabolic changes occur in chickens, specifically, broilers, reared in a HS environment, causing a considerable decrease in breast muscle size of the broiler chicken. HS is also responsible for the reduction in the protein content of muscles (14). Both AHS and CHS could cause a sharp decline in the metabolism of birds, which in turn will induce serious complications regarding the growth and performance of the broilers, such as a change in color, the decline in muscle pH, water-holding capacity (WHC), and juiciness of chicken meat (15, 16). Many studies have revealed that high ambient temperature causes oxidative stress by producing reactive oxygen species (ROS). ROS has severe implications on skeletal muscle development, as they are responsible for lipid peroxidation in muscles (17, 18). Thus, understanding the mechanisms underlying, the causes, and effects of HS and the strategies that can be put in place to curb or control such global menace, can be beneficial in solving the global food insecurity issues. This review dealt deep in analyzing the available information surrounding HS impact and the strategies to limit the unwanted implications of this threat. Figure 1 illustrates the physiological, metabolic and genetic changes amid HS and its relation to meat production and quality in chicken.

## Heat Stress in Broilers; How Does it Proceed?

Any foreign stimuli, which alters the normal biological and physiological mechanisms within living cells and threatens the living organism's survival, is referred as stress (18, 19). In broiler production, environmental stress is often caused by numerous factors, including ambient temperature, which severely compromises birds' normal physiology, leading to poor production efficiency and food safety (20). In animals, stress often manifests in three stages. Firstly, the recognition of external stress by the body is known as a state of alarm. Secondly, stress induces the immune mechanism in living cells; thus, if stress persists, the body tries to adapt to that new environment. Despite all resistance, if the body still fails to cope with that stress, it leads to the exhaustion stage (21). Every living organism responds to HS accordingly, depending on the intensity and duration of stress. Numerous studies reported a substantial reduction in feeding and walking duration (discrete values) of birds kept under HS conditions as heat-stressed birds spend most of the time in acclimatizing activities such as panting, drinking more water, and resting to cope with the HS (22).

The neuroendocrine system plays a very significant role in HS response by inducing the autonomic nervous system (ANS) that often regulates fight and flight situations in living organisms (23). In response to HS, the ANS takes charge and triggers tachycardia (increased heartbeat), increases respiration rate and enhances the blood flow toward the body peripheries (skin) for maximum heat loss to maintain body temperature (23, 24). It also promotes the breakdown of glycogen into glucose in muscles and reduces their capacity to store energy (6, 13). Activation of the neuroendocrine system positively regulates the release of catecholamine. Catecholamine acts on beta androgenic receptors of skeletal muscles and initiates a series of reactions, disturbing the normal enzymatic activity in skeletal muscles as it inhibits the enzyme glycogen phosphorylase and activates the muscle glycogenolysis (25). HS also activates the hypothalamic-pituitary-adrenal axis (HPA) along with the sympathetic-adrenal-medullar axis (SAM), which promotes the release of glucocorticoids, vasodilation, lipolysis, and proteolysis in muscles (26, 27). Glucocorticoids enhance glucose synthesis to confirm the survival of animals under such critical conditions as HS. The substantial release of glucocorticoids characterizes AHS as compared to CHS. Glucocorticoids encourage proteolysis by damaging myofibrils in skeletal muscles facilitated through major proteolytic mechanisms (ca^+2^ dependent, ubiquitin-proteasome system) (28, 29).

**Figure 2:**
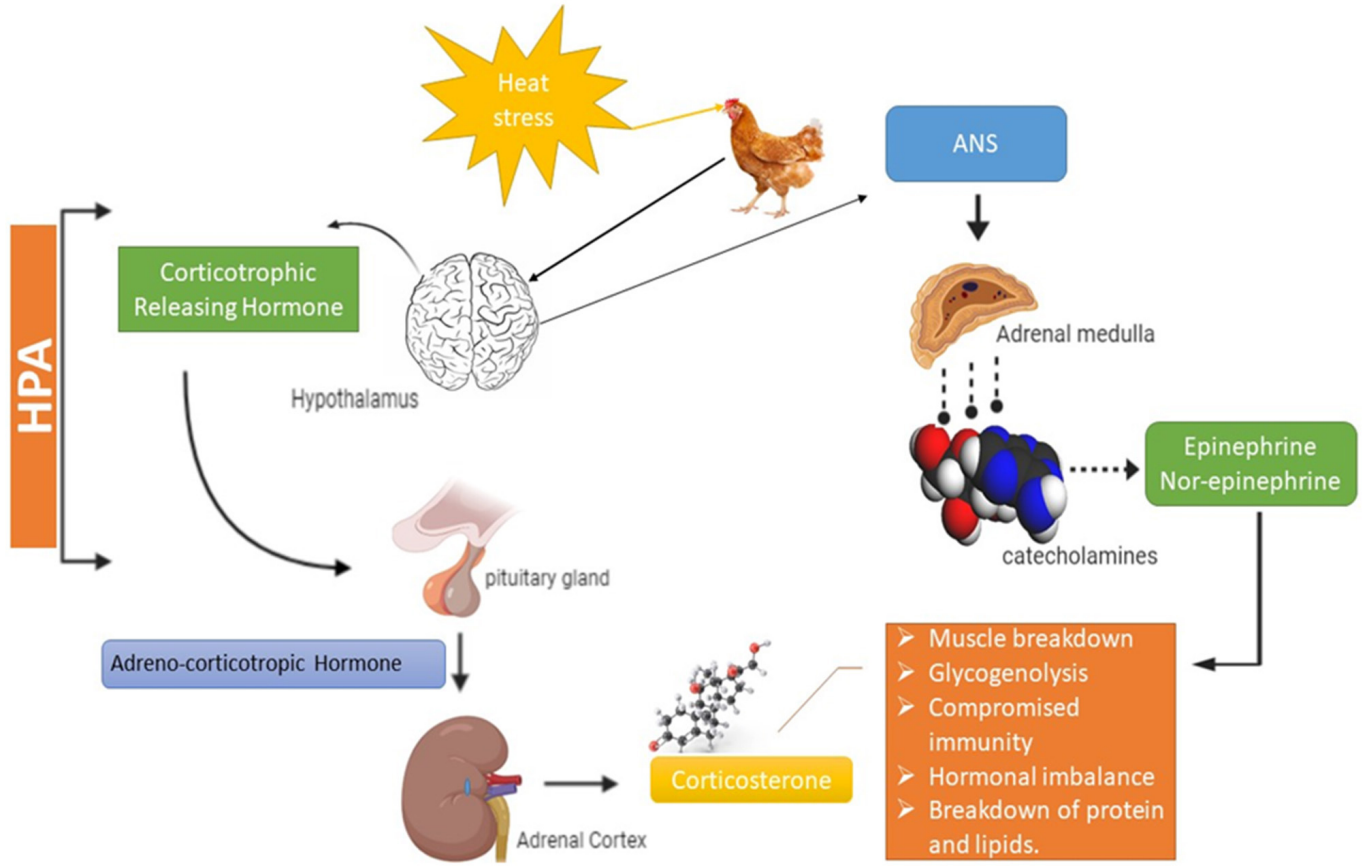
Mechanism of heat stress in broiler. HPA, Hypothalamic pituitary adrenal axis; ANS, Autonomic nervous system.

Furthermore, glucocorticoids initiate the hydrolysis of circulating triglycerides, intensifying the activity of lipoprotein lipase that leads to an increase in lipolysis. Moreover, anabolic factors like insulin growth factor (IGF-1) are negatively regulated by glucocorticoids to worsen the skeletal muscle damage. HPA is considered a better indicator of HS than corticosterone as it could be secreted in many other conditions like fear of invading animals etc. (30, 31). Corticosterone is secreted from both the HPA axis and the pituitary gland, corticosterone's secretion rate is relatively as slow compared to adrenaline but displays more compound and prominent effect during HS (32, 33). Long-term secretion of corticosterone during chronic HS is linked to many deleterious consequences in broiler chicken, including compromised immunity, muscle breakdown, cardiac issues, and depression (Figure 2). HS also induces infertility by disturbing reproductive hormones, severely affecting poultry gut health (leaky gut), as well as the altering of the immune functioning by triggering inflammatory cytokines (34).

## Thermoregulatory Apparatus in Chicken

All homoeothermic organisms have an optimal temperature range considered as the thermo-neutral zone unlike poikilotherms whose body temperature varies greatly depending on environmental temperature. In case the environmental temperature increases, the birds require more energy to maintain their body temperature (35). During HS conditions, metabolic heat increases, and animal succumbs to hyperthermia. Birds do not have sweat glands unlike mammals, but they have developed some behavioral adaptations to cope with heat, including elevated respiration rate, panting and raised wings (35, 36). In commercial poultry, high production always remained a priority that made the broiler more vulnerable to environmental stressors. The insulation provided by feathers in commercial poultry is one of the major hindrances in birds' thermoregulation (35, 37). To sum up, high ambient temperature beyond the thermo-neutral zone during the production phases badly affect meat production, meat quality and cause severe immune problems in the broiler flocks.

## Impact of HS on Poultry Meat Production

### Reduction in Feed Intake and Poor Weight Gain

Reduction in feed intake in HS animals is an adaptive mechanism to minimize metabolic heat production. A significant decrease in feed intake, body weight gain, and feed efficiency has been reported in many studies conducted on birds and other animals. During stress conditions, the priority of every living organism is to survive rather than growth. A recent study on broilers revealed that both cyclical and continuous heat stress significantly compromises growth performance by reducing protein digestibility up to 9.7%. Broilers under heat stress (32°C) have shown increased metabolizable energy intake (20.3%) and heat production (35.5%), and decreased energy retention (20.9%) and energy efficiency (32.4%) as compared to control group (38). Another study in laying hens reported a significant decrease in weight gain as average body weight (BW) of heat stressed hens was recorded 1.233 kg as compared to 1.528 kg BW of control group after 5 weeks of chronic heat stress (35°C). The significant decrease in weight is possibly due to reduced feed intake as birds under heat conditions ate less feed in relation to the control ones (39). This reduction in feed intake and nutrient digestibility severely compromises production efficiency and product quality. Chicken meat quality deteriorates since poor nutrients availability causes a sharp decline in muscle glycogen reserves, leading to dark, firm, and dry (DFD) meat (16).

### Increase in Fat and Reduction in Protein Contents of Poultry Meat

High ambient temperature disrupts normal lipid metabolism (lipolysis) by downregulating the enzymes involved in lipid breakdown resulting in more fat deposition and reduced protein content in muscles (40, 41). Many publications reported increased fat content in chicken under HS that seems to be an adaptive mechanism in birds as they store more energy in the form of fat to avoid further heat production during metabolism. A study conducted by Zhang et al. (14) reported that broiler birds raised under CHS (34–36°C) showed reduction in breast muscle mass (31.53%) and thigh muscle (11.17%) as compared to the normal control group. Considerable reduction in breast muscle mass was characterized by a significant change in chemical composition with higher fat quantity and lower protein concentration in muscles. Another study also concluded that cyclical HS (33°C for 9 h, 25°C for 15 h 1–42 days) in broiler reduced breast muscle weight by 16% (42). Lu et al. (43) reported higher intramuscular fat, increased activity of pyruvate kinase and lactate dehydrogenase in pectoral muscles of broilers under HS (32°C for 14 days). Moreover, CHS reduces the rate of aerobic metabolism by disturbing the mitochondrial functioning, decreasing aerobic metabolic activity and promoting glycolysis consequently leading to more fat deposition in muscles, which ultimately deteriorates meat quality (44). A study reported more fatness and low protein content in HS-broiler-chicken as compared to those maintained at the thermo-neutral condition (45). Production and quality losses in broiler chicken are not merely due to the reduced intake. Many other factors, including physiological, biochemical, and hormonal changes, are equally involved in all these losses to the poultry industry.

## Excessive Heat Burden Triggers Metabolic Stress That Deteriorates Meat Quality

### Excessive Production of ROS Impairs Meat Quality

Genetic modifications for rapid growth in broiler chicken has made the chicken more vulnerable to environmental stressors (17, 46). Oxidative stress is among the major stressors, which can potentially halt chicken growth, having severe consequences on the broiler's meat quality. Increased ROS liberation is potentially damaging as it aggravates the aging of muscles, protein degradation and inactivates the nuclear proteins, including DNA and RNA. HS induces ROS production by impairing mitochondrial function leading to reduced aerobic metabolism of fat and glucose and enhanced glycolysis, which ultimately results in poor meat quality characterized by low pH and high drip loss (47). Living tissues have many antioxidants to cope with oxidants, if the balance among antioxidants and oxidants disturbs and oxidants exceed a certain limit within the body, this condition indicates oxidative stress. Mostly oxidants are produced during cellular metabolism in the mitochondria of living cells. Cellular metabolism is not the only source of oxidants, some external sources, including feed comprised of oxidized lipids and fats, are responsible for producing reactive oxygen species (48). According to Mujahid et al. (49), leakage of electrons from the mitochondrial respiratory chain during oxidative phosphorylation is the main source of ROS. HS increases ROS production by compromising the electron transport chain's functioning, which is necessary for energy production in the muscles (50).

ROS changes calcium sensitivity by oxidizing the thiol groups in the ryanodine receptor and damages an enzyme sarcoendoplasmic reticulum Ca^+2^-ATPase (SERCA). This enzyme maintains calcium balance within the sarcoplasmic reticulum by removing extra calcium. Due to ROS, this system for calcium control collapses leading to overwhelming muscle contractions, culminating in muscle dystrophy (51–53). Numerous studies reported that oxidative stress leads to cell death and causes oxidation of protein and lipids, which ultimately deteriorates production efficiency and quality. Production of ROS in mitochondria leads to cellular oxidative stress, and it has severe consequences on physiological and behavioral characteristics in birds, which ultimately reduces the performance efficiency of the commercial meat birds. In short, oxidative stress lowers ATP production, creates calcium imbalance, and oxidizes several proteins within mitochondria along with mitochondrial membrane disruption (48, 53, 54).

### Acidosis Lowers Water Holding Capacity (WHC) and Damages Meat Texture

Rapid pH reduction in chicken muscle is also associated with HS, and it has severe implications on meat quality or texture. Multiple studies indicated HS to potentially reduce muscle pH leading to harmful changes in muscles (55). HS triggers anaerobic glycolysis within the muscles during and after slaughtering of the animal, thus, more H^+^ and lactic acid accumulate in the muscles due to hydrolysis of ATP during the anaerobic glycolysis. This result in a rapid drop in the pH of muscles leading to low water holding capacity which then develop into an abnormal condition called pale, soft, and exudative meat (56, 57).

### Thyroid Hormone Imbalance Under HS Impairs Skeletal Muscle Development

Thyroid hormone plays crucial role in the thermogenesis of avian via the thermoregulation by controlling metabolic heat production that is crucial to maintain normal body temperature. Tri-iodothyronine (T3) and tetra-iodothyronine (T4) enhance basal metabolism by modifying the mitochondrial function and assists skeletal muscles to acclimatize with a changing environment. Recent study regarding thyroid hormones in heat-stressed chicken found that high ambient temperature reduces both activity and size of thyroid. Lower level of thyroid hormone has observed in different studies conducted on heat stressed (38 °C for 24 h) quail (58) and domestic fowl (59). Thyroid hormones from external sources have also been observed to have lower survival time during HS (60). In broiler chicken, the thyroid gland's size, along with activity, decreases by high ambient temperatures and vice-versa (15). High ambient temperature normally responsible for drop in T3 and T4 plasma concentration. This mechanism is an adaptive tool to escape extra heat load, by decreasing metabolic heat production, plummeting maintenance energy requirements and increasing fat deposition by discouraging lipolysis (45, 61).

### How Does Meat Quality Deteriorate?

#### Drip Loss

After slaughtering, when muscle converts to meat, it loses some of its contents, including water, myofibers, iron, and proteins. Loss of muscle contents during which meat tends to lose its original texture and taste are often referred as drip loss (62). When frozen meat is being thawed, it loses its texture and taste due to loss of water contents and leakage of other nutritional contents through the water. Drip loss is related to overall meat quality as it reduces meat palatability, juiciness, and acceptability of meat. It is one of the major meat quality defects, of which experts are trying to resolve, most particularly in pork and chicken (63). HS before slaughtering of bird increases metabolic rate and rigor mortis that results in protein denaturation. As protein is involved in the water-binding capacity of meat, so protein damage due to high carcass temperature hinders protein ability to bind water that culminates into pronounced reduction in poor water-holding ability characterized by higher drip loss and cooking loss (64). According to a recent study, constant high temperature harms water-holding capacity because it increases drip loss in poultry meat. The researchers found that broiler birds' meat under high temperatures had increased value of cook loss, shear force, and decreased pH. Birds under cyclic heat had higher cook loss value in breast muscles as compared to those raised under the thermo-neutral environment (14). Practical observations and studies have demonstrated both AHS and CHS during the housing period of broiler to be responsible for poor water-holding capacity.

#### Development of Pale, Soft, and Exudative Meat

In chicken, the development of PSE is mainly due to the rapid decrease in pH after birds' slaughtering. Birds with high metabolic activities and efficient growth rates often have poor thermoregulatory ability; consequently, these birds are more prone to HS during the growing period (65). HS, during the broiler's growth period especially, causes multiple problems, including muscle atrophy, acid-base imbalance, and poor meat quality. In chicken, mostly muscles are comprised of fast twitching fibers. Fast twitching fibers are mainly dependent on anaerobic glycolysis (66). HS before slaughtering accelerates the anaerobic glycolysis in muscles and lowers pH during the conversion of muscles into the meat while the body temperature is high (67). High carcass temperature with low pH causes protein degradation and develops PSE condition (68). The processing capability of PSE meat is poor making processed meat more dry and brittle due to lack of proper WHC and protein extractability (69, 70). During hot weather, the broiler industry reports extensive losses in meat production due to reduced water holding capacity, poor meat texture, and pale color (57).

#### Production of Protein Carbonyls

AHS downregulates the protein synthesis at the transcriptional level, and it alters both ribosomal gene transcription and protein synthesis, consequently reducing protein deposition. The different durations of HS have different implications on the protein metabolism of hyperthermic animals (15). Short duration HS increases protein catabolism (marked by an increased plasma uric acid level), reduces protein synthesis and N retention, which decreases plasma concentrations of aspartic acid (Asp), serine (Ser), tyrosine (Tyr), and cysteine (Cys) (71). However, CHS knockdown protein synthesis in various muscles, decreases protein breakdown, with lower levels of plasma amino acids (especially sulfur and branched-chain amino acids) and higher serum levels of Asp, glutamic acid (Glu), and phenylalanine (Phe) (45, 72).

## The Genetic Basis of Muscle Development and Heat Stress

**Figure 3:**
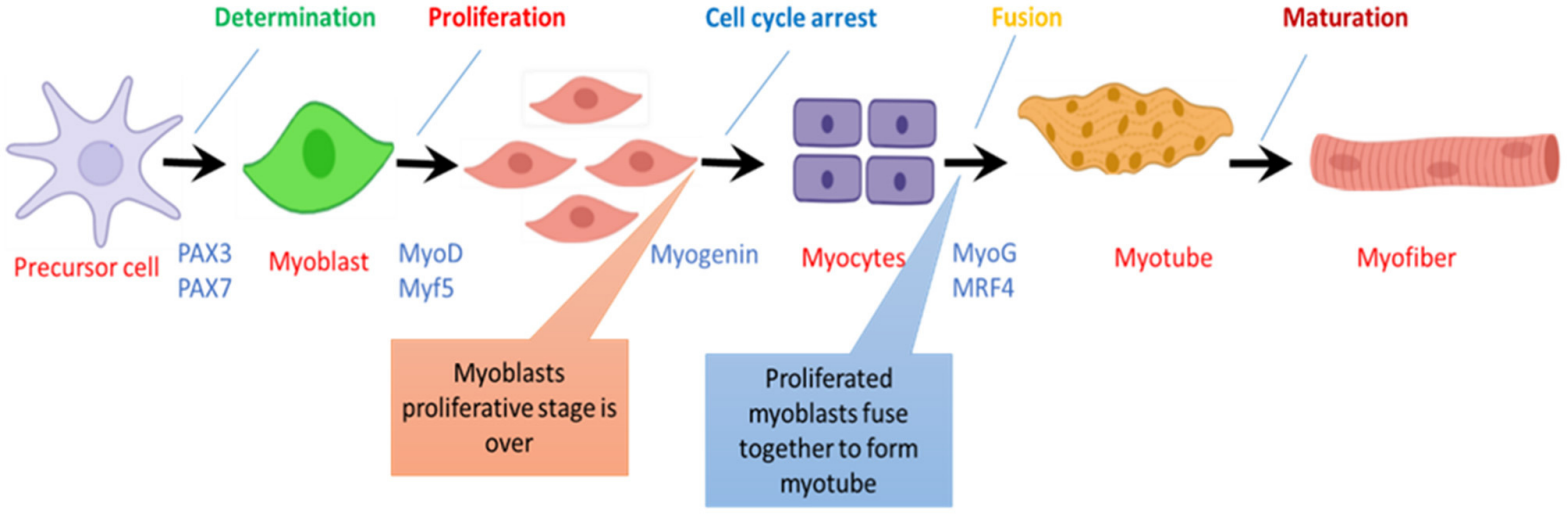
Step wise progress of myofiber formation during skeletal muscle development.in chicken.

Skeletal muscles contribute up to 40–60% of total animal body weight and play a crucial role in the movement, respiration, and homeostasis of the animal body (73). Moreover, they play significant role in the food industry and have significant economic importance. Especially in meat-producing animals, scientists and researchers are busy finding multiple ways to enhance skeletal muscle mass (74). Each muscle cell in skeletal muscle is termed as myofibril having multiple nuclei. This myofibril arises from the fusion of mesoderm progenitor cells called myoblast. In almost every major species, the number of myofibrils set at the time of birth and cannot be increased after birth, but muscle size can be increased (75). In chickens, muscle growth after birth is only due to hypertrophy, characterized by proliferation and fusion of activated satellite cells with muscle fibers and increased protein synthesis ability. Myogenesis is an intricate process having multiple steps determined by numerous myogenic factors including transcription factors, adhesion, molecules, growth hormones, and myogenic regulatory factors (76). Figure 3 illustrates the stepwise process of muscle cell formation and highlights genetic factors, which regulate the myofiber formation at every step.

Myogenic Regulatory Factors (MRFs), namely; Myf5, MyoD, myogenin, and MRF4, are members of the basic helix-loop-helix family of transcription factors that control the determination and differentiation of skeletal muscle cells during embryogenesis and postnatal myogenesis (77). MRFs form a family of transcription factors whose function and activity represent a paradigm where a series of molecular switches determine an entire cell lineage's fate. The MRFs are a group of muscle-specific proteins that act at multiple points in the muscle lineage to cooperatively establish the skeletal muscle phenotype by regulating the muscle cell proliferation, irreversible cell cycle arrest of precursor cells, followed by a regulated activation of sarcomeric and muscle-specific genes to facilitate differentiation and sarcomere assembly (78). A study on the mouse model has shown that MyoD, Myf5, and MRF4 are responsible for a myogenic determination as in the absence of these factors, there will be no skeletal muscle formation. At the same time, myogenin works as a differentiation factor. As myogenesis initiates, Myf5 is the first regulatory gene to be activated near the neural tube while Mrf4 is also activated during the early stages, but later on, it expresses only during the differentiation of the skeletal muscles (79, 80).

Constituents of the endocrine system, such as growth hormone (GH), IGF-1, and androgens, are the principal regulators of muscle metabolism. These endocrine components significantly impact muscle growth and act as anabolic factors, the major regulators of muscle's bulk (81). IGF-1 is considered to play key roles in fetal development and growth up to adolescence and in maintaining homeostasis in adult tissues by regulating cell proliferation, differentiation, and survival (82). IGF-1 exhibits a direct and crucial influence on muscle growth and differentiation during skeletal muscle development. Numerous studies have reported that IGF-1 is a positive regulator of myogenesis, which tightly controls the whole process of myogenic development. It is involved in various phases of myogenesis and muscle regeneration: triggering satellite cell proliferation, increasing protein synthesis, and promoting differentiation (82, 83).

MRFs are key regulators in skeletal muscle development, and numerous studies reported that HS has negative implications on myogenic regulatory factors. Low expression levels of MyoD, myogenin had been observed in chicken embryos at high temperatures (84). HS, during the embryonic development phase, postpones the formation of myofibers, consequently affecting the muscle proliferation and differentiation at later stages. The number of muscle fibers is fixed at the time of birth and can never increase; the only size of those fibers increases, and major muscle growth in chicken is carried out by hypertrophy lead by protein deposition (85, 86). A study on muscle development reported that HS impairs muscle hypertrophy by reducing the IGF-1 gene expression level and circulating IGF-1 concentration. HS also decreases the expression of MyoD, MyoG, and consequently hinders muscle hypertrophy by inhibiting S6K1. S6K1 plays a major role in cell growth regulation and muscle hypertrophy (84, 87). A study demonstrated that knockdown of S6K1 in rats caused a significant reduction in muscle size. This molecule is responsible for cell growth by increasing muscle cell size without affecting the cell number (84, 88).

To conclude, HS reduces growth performance, breast muscle mass, and yield in broilers. HS also reduced the mRNA expressions of IGF-1 and its downstream genes in breast muscle, thereby induced inactivity of mTOR and its downstream target S6K1 that regulates MRFs to decrease muscle hypertrophy. Meanwhile, the reduction of muscle protein synthesis is caused by reductions in both muscle amino acid uptake and the expressions of specific transporter isoforms due to the inactivity of mTOR and S6K1 (84).

## Heat Shock Proteins During Heat Stress

Heat shock proteins are widely considered as stress proteins found within the cells of all living organisms. During the high ambient temperature, living cells trigger a response named “heat shock response,” which activates the specific set of proteins to protect cells from stressors like heat (89). The primary function of heat shock proteins is housekeeping, they maintain order in the cell by synthesizing other proteins while during stressful environment or any pathological condition, their expression level increases and they incline to attract immune cells at the respective site or organ (90, 91). HSPs comprised six members classified and named on the basis of molecular weight, including HSP40, HSP70, HSP90, HSP100, small HSPs, and chaperonins. HSPs originate from an extracellular environment and function in specific parts of the body as stress signals and trigger immune cells during any stress and unfavorable conditions. HSPs also play an important role in protein formation and degradation by regulating folding/unfolding and translocation of proteins. All living organisms produce heat shock proteins under HS environment as these proteins are only produced under the stimuli of any stressor such as high temperature (91–94).

### Role of HSPs as Stress Indicator and Cell Protector

HSP70 is very crucial for cell recovery after the damage done by HS (95). Increased expression levels of HSPs during HS is an adaptive phenomenon that improves tolerance level against HS in living cells as the studies on the transcriptional behavior of heat shock proteins have revealed that HSPs are the heat polypeptides produced due to high temperature. HSP70 and 90 are more extensively studied families among heat shock proteins, and these two families exhibit a plethora of functions from involving in cell tolerance to control cell cycle (96–99). Exposure to high ambient temperature enhances the production of heat shock proteins, which are synthesized in respective cells experiencing stress, and helps synthesize other proteins. It also regulates many processes, including protein refolding, translocation, and prevents the oxidative breakdown and apoptosis of damaged proteins during stress conditions. All these functions carried out by Heat shock proteins are very handy for cell recovery after stress (96, 100–102). Studies revealed that HSPs play regulatory roles in various types of immunity. Production of HSPs during HS is mainly to attract immune cells. Numerous studies on differentially expressed genes during HS have shown that HSPs are related to birds' immune functioning during HS (103).

### HSPs Protect the Muscle Cells From Damage

In an HS environment, HSPs repair the damaged proteins. In normal climatic conditions, HSP 70 is present in low concentrations as molecular chaperones, while the level of HSPs increases rapidly in muscles during cellular stress (hyperthermia, oxidative stress, changes in pH). An increase of HSPs leads to significant changes in gene expression leading to remodeling of skeletal muscles (104). Numerous studies in broiler chickens reported that the HSP family is playing a key role to repair the damaged cells, and it has observed during acute stress, HSP70 expressed in the muscles, liver, heart, kidney, and blood vessels (101, 105). During AHS, an upregulated gene expression of HSP70 and 90 have been observed in muscle cells of broiler chicken. Moreover, AHS triggers both protein and mRNA expression of HSP70 and 90 in the kidney of chicken. A study conducted on Taiwanese roosters under acute HS has revealed the upregulation of HSP70 and 90 in Taiwanese roosters' testes. In contrast, another study reported depression in the expression level of HSP 90 and HSP25, which are believed to be involved in protein folding (101, 106, 107).

### HSPs Regulates Meat Quality by Inhibiting Muscle Apoptosis

After the slaughtering of animals, muscles undergo cell apoptosis due to the unavailability of oxygen and nutrients within muscle cells. All those factors involved in the apoptotic activity of muscle cells are considered to control the animal's ultimate meat quality. Multiple studies reported the role of small heat shock proteins as an anti-apoptotic factor in muscle cells during post-mortem changes in muscles of slaughtered animals and influences the meat quality attributes including color, tenderness, juiciness, and the meat flavor (108, 109). After the muscle undergoes cell death due to apoptosis, the number of small heat shock protein increases at that side and lowers the rate of apoptosis and unfolding of proteins in muscles (110). They delay protein degradation in muscle cells and try to maintain homeostasis at the cellular level. In this way, small heat shock proteins impede the aging process and play a crucial part in developing meat quality (110, 111).

## Dealing With HS to Improve Meat Production and Meat Quality

### Dietary Supplementation

Multiple nutritional strategies have been suggested to alleviate HS destructive effects in the poultry industry. Previous studies revealed that protein metabolism is severely affected by chronic HS and leads to reduced protein deposition in muscles. This dwindling protein level cannot be compensated through dietary protein because it further aggravates HS by producing more metabolic heat (112, 113). On the other hand, reducing protein concentration in diet culminates in to poor weight gain and lower feed efficiency. Chickens on a low protein diet often consume more feed to fulfill their protein requirements and the consumption of more feed results in poor feed efficiency. It has suggested that feed with more fat supplementation and low protein contents could minimize HS mischievous impact (114, 115). A similar study (116) proposed that feed supplemented with 5% fat and 4% palm oil can improve broiler production performance under the HS environment by lowering feed retention and optimizing the nutrient utilization. Secondly, Feed restrictions during the early period of life in chicken have been proved handy in reducing HS's damaging effects. A study demonstrated that feed restriction during early days of broiler chicken (4–6 days after birth) promotes heat tolerance later in life (35–40 days of age) (7, 116). Early feed reduction (EFR) and fat supplemented feed have a beneficial impact on heat-stressed broiler birds.

Thirdly, ample supplementation of vitamins is obligatory for better broiler production, especially amid harsh environment (10, 116, 117). Vitamin supplementation through drinking water is common practice in some poultry farms that have proved helpful to boost immunity and enhance heat-stressed broilers' performance. Diets containing vitamin A help broilers to fight against oxidative injuries induced by high environmental temperature (118). Kucuk et al. (118) also reported that vitamin A fortification has positive effects on production status as it enhances body weight gain, feed efficiency and reduces oxidative damage. Poultry birds can synthesize Vitamin C by itself and does not seek an external supply of vitamin C during normal conditions. However, under stress conditions, the additional supplementation of vitamin C might be fruitful for broilers' better performance as it promotes fatty acid oxidation instead of protein breakdown and reduces respiratory quotient (119). Studies reported increased hunger of birds for vitamin C during HS as vitamin C promotes fatty acid oxidation instead of protein breakdown and reduces respiratory quotient (119, 120). Moreover, it enhances meat quality by producing meat with high protein and low-fat contents and maintains redox status during high temperature because of its ability to be one of the best antioxidants. A study based on vitamin E diet supplementation reported that vitamin E supplementation promotes the phagocytic activity of macrophages and increases serum antibodies (IgM and IgG) levels in broiler under HS (121).

#### Use of Herbs

There has been much attention placed on how herbal feed additives can be used in alleviating the adverse effects of HS, which in a way will help to enhance the production and performance of other animals, including poultry, pigs, and rabbits (122). The advantages of herbal additives include pharmacological and nutritional values and amelioration of many animal diseases. For example, there was noticeable recovery reported in animals which suffered harmful HS sequence after dietary supplementation of some herbs such as Ginger, Fennel, Black seed, hot red pepper, *Artemisia annua*, Rosemary, Moringa, *Radix bupleurum*, Chicory, and Dill (123).

Ginger (*Zingiber officinale*) as widely known to be used in the treatment of lots of disorders (119), contains compounds such as gingerdione, gingerdiol, and shagaols, which possess quite a lot of antioxidant and antimicrobial activities (119, 124). The addition of ginger (2%) to heat-stressed broilers significantly improved the biochemical blood parameters and the growth performance in comparison to the control whereby the changes which emanated were attributed to antibacterial potential of the supplement, which in effect improved the digestibility, palatability, metabolism, and health status of the chicken (119, 124). HS is noted to affect the poultry by reducing the villus height in quail (125) and broiler chicks (126). However, broilers supplemented with 2 and 4 g/kg garlic diets revealed the highest intestinal villi and most significant crypt depth in comparison to the control as reported by Shewita and Taha (127) although negative impacts on body weight, FCR, and FI at higher levels (6 g/kg) were reported. A report by Khonyoung et al. (128) showed that dietary supplemented with fermented-dried ginger products at 1% can help reduce abdominal fat, which in effect can help improve the health of heat-stressed broilers. For Fennel (*Foeniculum vulgare*), lots of research showcases the role that its essential oil plays as an antioxidant, antimicrobial, and a potent hepatoprotective agent (129, 130). A study conducted by Ragab et al. (131) revealed an improvement of feed intake, meat breast (%), and leukocytes of heat-stressed Ross broilers after 1 or 2% of this herb. Correspondingly, fennel fruits supplementation at 10 or 20 g/kg diet in heat-stressed laying hens significantly improved the quality of eggs, reduced the malondialdehyde (MDA) contents, carboxyl levels in eggs, and again reduced the triglyceride and cholesterol contents (132). Again, Mohammed and Abbas (133) also observed that feeding of chicks with 1, 2, and 3 g fennel/kg diet significantly increased the RBCs, Hb, and PCV in comparison to the control.

Again, *Nigella sativa*, commonly known as the black seed, has been used in many HS research of poultry, and effect has shown encouraging results due to the higher nutritional values it carries. Active materials such as thymoquinone, nigellone, and thymohydroquinone, which aids in exerting antitoxic and antimicrobial properties through increased defense mechanisms against infectious diseases, are reported to be contained in black seed (134). Heat stressed pigeon which was fed with 2% black seed aided in weight gain and body weight improvement, hepatic lesion protection, which led to mild vascular congestion and vacuolization of the hepatocyte without creating damages to sinusoids in comparison to the control [EL (135)]. Heat stressed broilers subjected to a 1% black seed diet increased the feed intake, dressing percentage, body gain while reducing the panting behavior, water to feed ratio, corticosterone, and T3 levels (136). Judging from these, ginger, fennel and black seed herb among others can be used to reduce the bad effects HS is noted to have on the poultry production.

#### Probiotic Effects on HS in Poultry

The supplementation of feed additives such as probiotics, prebiotics, and symbiotics has been used lately to curb the negative impacts HS poses in birds (9). Probiotics are “live microorganisms which, when administered in adequate amounts, confer a health benefit on the host (137).” Lots of research have been conducted proving that probiotics administration in diets is a sure way of improving the growth, immune response, digestive enzyme activity, disease resistance, gut microbiota in aquatic animals (138–141) chicken (142), pigs (143), etc. Given this, probiotics have gained lots of attentions from scientists in the poultry industry as the addition of this additive is a sure way of enhancing the intestinal morphology, physiological conditions, immunity; thus, the overall well-being and performance of heat-stressed poultry as previously reported (144, 145). A study conducted by Zulkifli et al. (146) reported that a probiotic-enhanced water acidifier (*S*. *faecium* and *L*. *acidophilus* + citric acid + sorbic acid + sodium citrate + sodium chloride + zinc sulfate + ferrous sulfate + potassium chloride + cellulase +magnesium sulfate) aided in the restoration of Na and K levels in broilers after 1 day HS. Broilers subjected to HS saw an increase in T_3_ (147) and T_4_ (148) in the serum after administering probiotics.

There has been a report that “Protexin^®^ Boost,” a probiotic treatment, improved serum uric acid levels of heat-stressed birds. Uric acid plays a critical role as an antioxidative agent (149); thus, an increase in its level after the probiotic treatment depicts that the probiotic exerts some mechanism in alleviating the oxidative damage after the HS in birds. Hasan et al. (150) observed an increase in hemoglobins in heat-stressed birds after dietary supplementation of probiotics (Protexin^®^ Boost). Furthermore, probiotics have also been revealed to improve the immune system of HS birds (151). It has been established that, the administration of probiotics enhances not only the responses of antibody (146, 151, 152) but also leukocytes count (153) in heat-stressed birds. Intraepithelial lymphocyte (IEL), an important host immune system component, is noted to respond rapidly when host organisms are infected (154). An experiment executed by Deng et al. (151) revealed a lower IEL number in the cecum and ileum of laying hens at week 61. Hasan et al. (155) revealed that the lymphoid organ's involution due to HS in poultry could be prevented by *B*. *subtilis* supplementation. Correspondingly, Lei et al. (156) observed a reduction in the corticosterone levels, which causes lymphoid organ involution after HS. Studies show that probiotics' dietary supplementation enhances the intestinal composition after HS conditions (144). Many studies on the health and well-being of heat-stressed poultry after supplementation have been established, as some have been discussed. Table 1 also enlists other research performed previously, which reveals the positive effects of probiotics in improving microbiota, morphology, and immune response of heat-stressed poultry.

**Table 1:** Role of different probiotics to counter the damaging impact of heat stress in poultry.

**Probiotics name**	**Poultry strain**	**Findings which aided in countering heat stress**	**Country of Investigation**	**Sample size**	**References**
Multi strains probiotics (*L*. *plantarum,* *L*. *bulgaricus,* *L*. *acidophilus,* *L*. *rhamnosus,* *B*. *bifidum,* *S*. *thermophiles)*	White layer (Hy-line variety)	(1) Strengthens antibody titer against SRBC	Iran	60	(157)
Probiotic *B*. *licheniformis*	Hy-line Brown	(1) Enhanced mucosal immunity (IgA-secreting cells) in heat-stressed chicken (2) Overturned the increased levels of serum TNF-α and IL-1 due to HS(3) Improved IEL counts in the ileum and cecum of heat-stressed chicken(4) Counter the increased number of mast cells in the ileum and cecum of birds due to HS	China	96	(151)
Probiotic mixture (*L*. *pentosus* ITA23 and *L*. *acidophilus* ITA44)	Broiler chicken (Cobb-500)	(1) Improved antioxidant ability of liver in chicken raised at high ambient temperature (2) Improved the population of following bacteria in heat-stressed chicken a. *Bifidobacteria* b. *Lactobacillus* c. *Enterococcus*	Malaysia	192	(145)
*B*. *subtilis* and *B*. *licheniformis*	Duck (cherry valley pekin Ducks)	(1) Augmented expression levels and enzyme action of LXRα, which wheels the functional specialty of splenic macrophages in ducks	China	750	(158)
Probiotic *S*. *cerevisiae*	Broiler chicken (Cobb-400)	(1) Enhanced the villus height in the duodenum of broilers raised under HS (2) Reduced the number of *Salmonella* and *E*. *coli* in excreta and gut of HSed broilers	Turkey	175	(152)
Probiotic mixture (*L*. *acidophilus, L*. *casei, E*. *faecium*, and *B*. *bifidium*)	Broiler chicken (Ross-308)	(1) Improved antibody responses to Newcastle disease (ND), Bronchitis, and Gumboro disease in broilers under cyclic HS	Iran	96	(159)
Lactobacillus sp. and yeast culture	Arbor Acres broiler	(1) Reduced the population of *E*. *coli* and *Salmonella pullorum* (2) Lessened the pH of the intestine (duodenum, jejunum, ileum, and cecum) in a heat-stressed broiler		300	(147)
Lactobacillus-based probiotics (*L*. *plantarum*, *L*. *acidophilus*, *L*. *bulgaricus*, *L*. *rhamnosus*, *B*. *bifidum*, *S*. *thermophilus*, *E*. *faecium*, *A*. *oryzae*, and *C*. *pintolopesii*)	Broiler chicken (Hubbard)	(1) Regained villus height and crypt depth in duodenum and ileum of a heat-stressed broiler (2) Maintained the activity of goblet cells	Pakistan	250	(160)
Probiotic *B. subtilis*	Hubbard broiler	(1) Improved the population of useful Intestinal bacteria (*Lactobacillus* and *Bifidobacterium*) (2) Renovated the reduced villus-crypt structure	Jordan	480	(144)
Probiotic mixture (*B*. *licheniformis, B*. *subtilis*, and *L*. *plantarum*)	Ross-308	(1) Improved the viable counts of small intestinal *Lactobacillus* and *Bifidobacterium*, and reduced coliforms in a heat-stressed broiler (2) Enhanced villus height in the jejunum and improved intestinal barrier function	China	360	(161)
Lactobacillus-based probiotics (*L*. *plantarum*, *L*. *acidophilus*, *L*. *bulgaricus*, *L*. *rhamnosus*,	Hubbard	(1) Ameliorated the inflammatory response (decreased excessive numbers of IEL) in all intestinal segments of heat-stressed broilers (2) Increased the count of goblet cells in the intestine (duodenum and jejunum) of heat-stressed broilers	Pakistan	250	(160)
*B*. *bifidum*, *S*. *thermophilus*, *E*. *faecium*, *A*. *oryzae*, and *C*. *pintolopesii*)					
Probiotic mixture (*L*. *plantarum*, *L*. *delbrueckii* ssp. *Bulgaricus*, *L*. *acidophilus*, *L*. *rhamnosus*, *B*. *bifidum*, and *S*. *salivarius* ssp.)	Ross-708	(1) Developed intestinal microarchitecture (villus width and surface area) of heat-stressed broilers	United States	450	(162)

*IEL, Intraepithelial lymphocyte*.

### Introducing Heat Tolerant Traits From Indigenous Breeds Into Commercial Breeds

Introduction of new technologies such as genomics provides valuable data and new approaches to address these challenges. The commercial broiler industry's focus largely remained only on fast weight gain and feed efficiency from previous two decades. Commercial breeds capable of gaining more weight in thermo-neutral conditions when raised under a high-temperature environment fail to maintain their growth performance (15). Genetic selection for heat tolerance in broilers needs to be taken into account, especially in tropical and subtropical regions of the world. A specific phenotype “frizzled feather,” characterized by curly feathers waving outside, was reported by Darwin (163). It was proposed that this type of chicken gives the best protection against the severe environment and the specific gene revealing such characteristics expresses in many chicken breeds (164). A study reported that 69-bp deletion in KRT6A was responsible for frizzle character in chicken. On the other hand, our research group conducted study on local Chinese frizzle breed found a 15-bp deletion in the KRT75L4 gene (165). This natural mutation in the chicken genome is reflected as an adaptive mechanism as these birds can tolerate heat better and are mostly found in warm regions. Data on the country-wise distribution of different animal breeds on the FAO website revealed both naked neck and frizzled feather chicken found worldwide. The naked neck gene has also been observed to withstand extreme climatic changes like high temperature (116).

**Figure 4:**
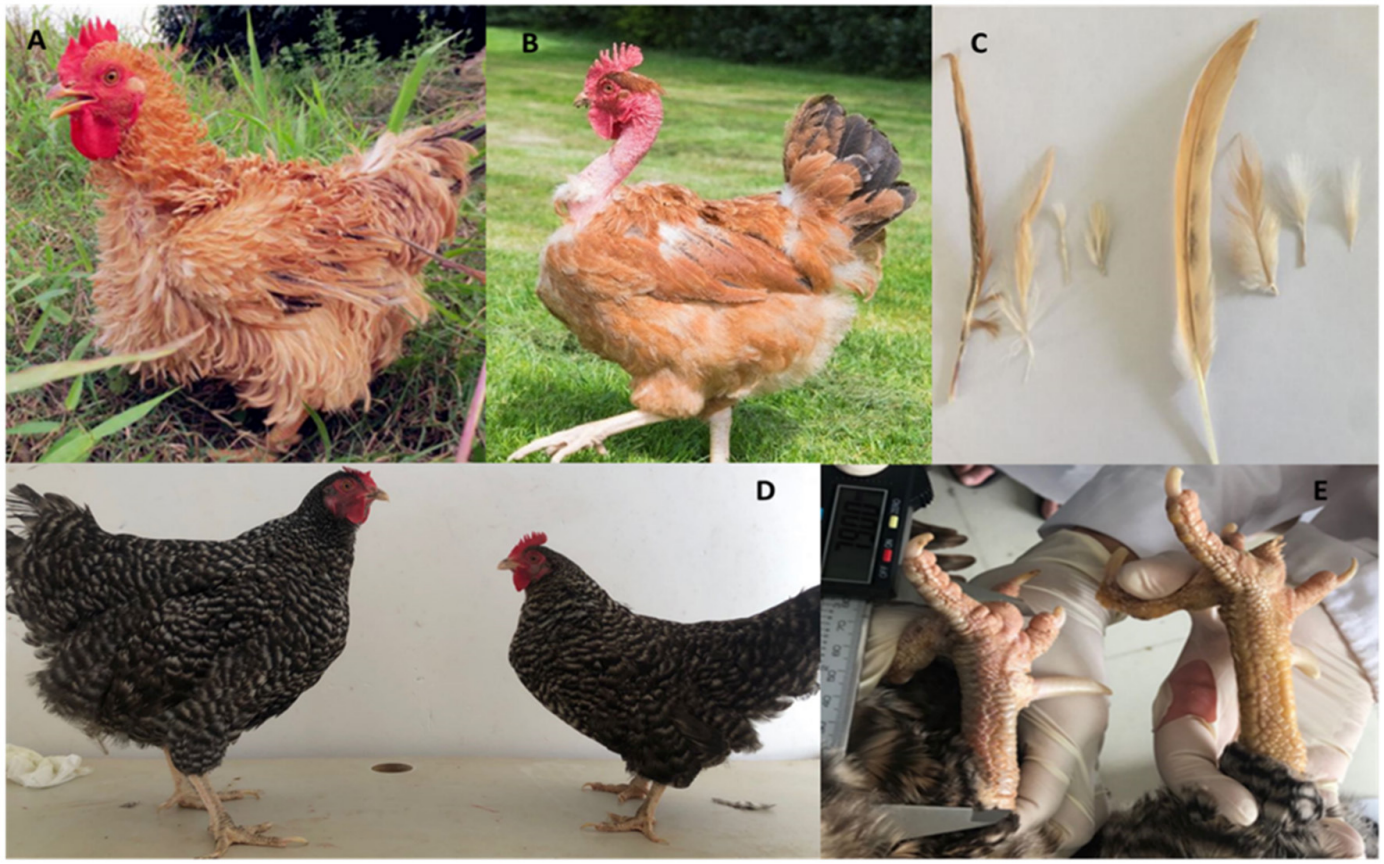
**(A)** Frizzled feather chicken **(B)** Naked neck chicken **(C)** Comparison among normal and frizzle feathers, frizzle feathers on the left side, normal feathers on right side **(D)** Dwarf size plymouth rock chicken with normal Plymouth rock chicken **(E)** Shank length of dwarf chicken as compared to normal chicken (These pictures have been taken in Guangdong Ocean University, Zhanjiang, China by our research group).

Naked neck (Na), Frizzle (F, candidate gene: KRT6A and KRT75L4), and Dwarf (Dw, candidate gene: GHR) genes in poultry are considered candidate genes to tolerate thermal stress. Naked neck gene reduces the feather mass up to 40% and lowers the chances of heat insulation due to more feathers on the skin (166, 167). Studies reported that Na chicken perform better under heat stress compared to birds with normal feathers. Better immunity and production performance have also been observed in the Na chicken line (168). Lack of feathers on the neck provides more space for heat dissipation and discourages heat insulation, helping birds tolerate the harsh temperature. Na gene has a considerable positive role in production performance and immunity development in birds. It also minimizes the fat deposition in the breast region, promoting heat dissipation, leading to heat tolerance (166, 169, 170). The dwarf (GHR) gene is also considered a heat-tolerant gene as it reduces body size from 30 to 40%. Na, F, and Dw genes could prove beneficial for the commercial poultry industry in tropical and subtropical parts of the world (171). Figure 4 shows different heat-tolerant breeds, including naked neck, frizzled feather, and dwarf chicken.

## Conclusion

With time, HS issue is becoming more challenging for poultry industry. Genotype selection in broiler birds for higher growth rates to meet ever-increasing food requirement has made broiler chicken vulnerable to HS. Unluckily, the detrimental consequences of heat stress for poultry health and production are likely to continue and to be acquired by next generation during gestation if selection for only production traits is prioritized against heat tolerance and climate adaption according to current trends of global warming. High producers, commercial broiler breeds cannot withstand HS resulting in substantial economic losses to the industry, which triggers food security issues. Genetic selection for heat tolerance in poultry is the only durable solution to curb HS's negative implications. Realizing this threat to food security, scientists and industry's concerted efforts will be required to overcome this problem. These efforts should include (a) Genotype profiling of heat-tolerant breeds along with comprehensive studies on the interaction between genotype and phenotype in both heat tolerant and susceptible broiler breeds. (b) To explore the complete molecular mechanism of muscle development and muscle growth during HS environment. (c) Crossing frizzled feathers chicken breed to dwarf breed may give more apparent illustration about molecular and genetic mechanisms underlying heat resistance. Apart from breeding strategies, adopting modern managerial and environmental strategies could minimize the deleterious effects of heat on meat production and quality.

## Author Contributions

AHN did the majority of the writing by communicating with KA and coordinated the document editing. LZ provided advice on the research input to the review article and performed significant edits to the document as well the funding acquisition. QYL, JHZ, and WLZ helped to gather and analyze information regarding the topic of review.

## Conflict of Interest

The authors declare that the research was conducted in the absence of any commercial or financial relationships that could be construed as a potential conflict of interest.

## Publisher's Note

All claims expressed in this article are solely those of the authors and do not necessarily represent those of their affiliated organizations, or those of the publisher, the editors and the reviewers. Any product that may be evaluated in this article, or claim that may be made by its manufacturer, is not guaranteed or endorsed by the publisher.
